# Characteristics, treatment and care of pregnant women living with hepatitis B in England: findings from a national audit

**DOI:** 10.1017/S0950268823000225

**Published:** 2023-03-02

**Authors:** Heather Bailey, Eleni Nastouli, Sharon Webb, Catherine Peckham, Claire Thorne

**Affiliations:** 1 UCL Institute for Global Health, University College London, London, UK; 2 UCL Great Ormond Street Institute of Child Health, University College London, London, UK; 3 Advanced Pathogen Diagnostics Unit, University College London Hospitals NHS Foundation Trust, London, UK; 4 Department of Clinical Virology, University College London Hospitals NHS Foundation Trust, London, UK; 5 NHS Infectious Disease in Pregnancy Screening (IDPS) Programme, Public Health England, London, UK (at time this work was completed); current affiliation: NHS England

**Keywords:** Antenatal care, England, hepatitis B virus, migrant health, pregnancy

## Abstract

Around 0.4% of pregnant women in England have chronic hepatitis B virus (HBV) infection and need services to prevent vertical transmission. In this national audit, sociodemographic, clinical and laboratory information was requested from all maternity units in England for hepatitis B surface antigen-positive women initiating antenatal care in 2014. We describe these women's characteristics and indicators of access to/uptake of healthcare. Of 2542 pregnancies in 2538 women, median maternal age was 31 [IQR 27, 35] years, 94% (1986/2109) were non-UK born (25% (228/923) having arrived into the UK <2 years previously) and 32% (794/2473) had ⩾2 previous live births. In 39%, English levels were basic/less than basic. Antenatal care was initiated at median 11.3 [IQR 9.6, 14] gestation weeks, and ‘late’ (⩾20 weeks) in 10% (251/2491). In 70% (1783/2533) of pregnancies, HBV had been previously diagnosed and 11.8% (288/2450) had ⩾1 marker of higher infectivity. Missed specialist appointments were reported in 18% (426/2339). Late antenatal care and/or missed specialist appointments were more common in pregnancies among women lacking basic English, arriving in the UK ⩽2 years previously, newly HBV diagnosed, aged <25 years and/or with ⩾2 previous live births. We show overlapping groups of pregnant women with chronic HBV vulnerable to delayed or incomplete care.

An estimated 296 million people are living with chronic hepatitis B (CHB) virus infection [1], a main cause of liver cirrhosis and hepatocellular carcinoma worldwide [2, 3]. Despite recent declines in incidence of CHB infections and age-standardised death rates for hepatitis B virus (HBV) cirrhosis [3, 4], global deaths from HBV-related causes remained at an estimated 820 000 in 2019, with many gaps in prevention, diagnosis and treatment that need to be addressed to achieve the World Health Organization (WHO) goals of elimination of viral hepatitis as a public health threat by 2030 [5].

Globally, around two-thirds of people with CHB live in the WHO African and Western Pacific regions, where hepatitis B surface antigen (HBsAg) prevalence was >5% in 2016 [6]. In high prevalence settings, most infections are acquired vertically or in early childhood [7], at which point over 80% of infections will become chronic *vs.* 50% if acquired in early childhood and 5% if acquired by immunocompetent adults [8]. Prevention of vertical transmission (VT) is therefore a key priority [9]. Without interventions, risk of HBV VT is 10–40% among women of lower infectivity and 70–90% among those of higher infectivity [10]. Birth dose of HBV vaccination with completion of the vaccination schedule in the first year is the mainstay for preventing HBV VT. Scale-up of infant vaccination coverage to 85% had reduced prevalence of HBV among <5 year olds to an estimated 0.94% worldwide in 2019 [5], with a further Global Health Sector Strategy target to reduce this to <0.1% by 2030 [11].

In England, around 0.4% of 700 000 women entering antenatal care (ANC) each year have CHB [12]. HBV screening is offered to pregnant women through the NHS Infectious Diseases in Pregnancy Screening (IDPS) Programme; coverage is currently 99.8%, up from 97.7% in 2014 [12, 13]. Babies identified as HBV-exposed receive HBV vaccine at birth, 1 and 12 months in addition to routine primary immunisations; passive immunisation with hepatitis B immunoglobulin (HBIG) is also given at birth to babies born to women of higher infectivity [14]. Use of active and passive immunoprophylaxis can prevent VT in around 95% of cases overall [10]. Since 2013, UK NICE guidelines have recommended treatment with antiviral tenofovir disoproxil (TDF) in the third trimester for women with HBV DNA level >10^7^ IU/ml, to further reduce VT risk [15] while the European Association for the Study of the Liver (EASL) has recommended TDF for women with a viral load (VL) >200 000 IU/ml since 2017 [16].

We present findings of a national audit undertaken on the management of pregnant women with HBV who initiated ANC in England in 2014. The audit aimed to inform strategies to prevent vertically acquired HBV infection and findings fed into the development of Public Health England (PHE) guidance on the HBV antenatal screening and selective neonatal immunisation pathway [17] and clinical guidelines from the British Viral Hepatitis Group [18]. Here, we describe maternal and pregnancy characteristics of the audit population, and indicators of access to/uptake of antenatal and specialist care.

## Methods

The Hepatitis B in Pregnancy Audit was a national audit of pregnancies in HBsAg-positive women initiating ANC in England from 1 January to 31 December 2014. Sociodemographic, clinical and laboratory information was requested from named ‘respondents’ (mainly antenatal screening coordinators) at all maternity units in England, following methodology modelled on the National Study of HIV in Pregnancy and Childhood [19] and the Surveillance of Antenatal Syphilis Screening (SASS) study [20].

Maternity unit respondents were asked to provide information securely via web forms and NHSmail at three time points for each pregnancy: antenatal booking (notification form), following specialist referral (referral form) and after pregnancy outcome/delivery (outcome form). Notification forms were completed on a rolling basis, with periodic prompts sent to respondents for submission of referral and outcome data on notified pregnancies.

Information collected for all eligible pregnancies included country of birth and family origin, languages spoken, obstetric information (history and current pregnancy), HBV diagnosis and test results, referral for and attendance at specialist care for HBV, pregnancy outcome and receipt of interventions to prevent VT. Additional data were collected for pregnancies of higher infectivity, defined as those with ⩾1 of the following test results reported in an antenatal sample at notification: HBeAg-positive; HBeAg-negative and anti-HBe-negative; HBV DNA ⩾1 × 10^6^ IU/ml [21], including investigations and obstetric complications during pregnancy and additional detail on delivery and the infant. For pregnancies of higher infectivity at notification and those where HBIG was later reported to have been ordered or given to the infant, additional information was requested from clinical specialists at >1 year postpartum. Pregnancies reported from more than one Trust were de-duplicated using NHS number (no names were collected); NHS numbers were also used to seek outcome information for women who were known to have transferred to another Trust within England before delivery.

Maternal country of birth was categorised into regions and sub-regions according to the United Nations classification system [22]. Family origin categories were taken from the NHS Sickle Cell and Thalassaemia Screening Programme Family Origin Questionnaire [23]. Woman's date of birth was collected to the month and year, with the 15th of the month used to calculate maternal age, defined as age at estimated date of delivery. English language levels were defined as fluent (sufficient to allow woman to cope with almost anything that she may encounter in the healthcare system), basic (speaks and understands English to a limited degree, probably sufficiently to allow her to be interviewed directly, but ideally in the presence of someone who can help to translate) and less than basic (unable to cope with appointments and literature without help from someone who can translate). ‘Late’ antenatal booking was defined as booking for ANC at ⩾20 gestation weeks. This cut-off was chosen due to the 8-week maximum duration between booking and specialist appointment specified in the 2010 IDPS programme standards [24], and the fact that women booking after 20 gestation weeks may therefore not access specialist care in time to initiate TDF at the start of the third trimester.

Maternal characteristics were compared by timing of ANC initiation (<20 or ⩾20 gestation weeks) and by whether ⩾1 specialist HBV appointment during pregnancy was known to have been missed, in order to identify groups with barriers to antenatal and specialist HBV care. Comparison of proportions was carried out using the *χ*^2^ test for categorical variables; differences in continuous variables were compared using the Wilcoxon–Mann–Whitney rank-sum test.

Data collection was managed using REDCap (Research Electronic Data Capture) forms [25] and managed and analysed using STATA version 15.1 (Stata Corp. LP, College station, TX, USA).

The audit had approval from the Secretary of State's Confidentiality Advisory Group (CAG 5-07(b)/2013). Although consent was not required, women were able to opt out of having their data included in the audit.

## Results

Of a total 2542 pregnancies in 2538 HBsAg-positive women reported to the audit across 128 NHS Trusts, 45% (1142) initiated ANC in the London region, 24% (602) in Midlands and East, 18% (457) in the North and 13% (341) in the South. One Trust declined participation and 64 HBsAg-positive women across 19 Trusts were known to have opted out of having their data included.

Median maternal age was 31 [IQR 27, 35] years (categories shown in Table 1) and two-thirds had at least one previous live birth. Overall, around half (130/239) of women in the <25 years age group had no previous live births compared with 43% (337/781) of 25–29 year olds, 31% (236/762) of 30–34 year olds, 19% (90/485) of 35–39 year olds and 16% (25/161) among those aged ⩾40 years (*χ*^2^ = 153.15, *P* < 0.01).

**Table 1.** Characteristics of HBsAg-positive women

	*n* (%)
*Maternal age (years) (n* *=* *2491)*	
<25	244 (10)
25–29	799 (32)
30–34	785 (32)
35–39	499 (20)
⩾40	164 (7)
*Previous live births (n* *=* *2473)*	
0	831 (34)
1	848 (34)
⩾2	794 (32)
*English language level (n* *=* *2120)*	
Fluent	1298 (61)
Basic	386 (18)
Less than basic	436 (21)
*Region of origin (n* *=* *2109)*	
Africa	650 (31)
Middle Africa	69 (3)
Eastern Africa	184 (9)
Northern Africa	13 (0.6)
Western Africa	374 (18)
Southern Africa	10 (0.5)
Americas (North America, Latin America and the Caribbean)	13 (0.6)
Asia	735 (35)
Central Asia	6 (0.3)
Eastern Asia	311 (15)
Western Asia	51 (2)
Southern Asia	254 (12)
South-eastern Asia	113 (5)
Europe	704 (33)
Eastern Europe	413 (20)
Northern Europe	72 (3)
Western Europe	129 (6)
Southern Europe	90 (4)
Oceania	7 (0.3)
*Timing of arrival into UK (n* *=* *923)*^a^	
>10 years ago	181 (20)
5–10 years ago	308 (33)
2–4 years ago	206 (22)
<2 years ago	228 (25)

a Available for 46% (923/1986) of women born outside of the UK.

Among 83% (2109/2538) women with country of birth reported, this spanned 104 different countries with around a third (650) born in Africa, a third (735) in Asia and a third (704) in Europe (Table 1); the most common countries of origin were China (14%, 288), Romania (10%, 211), Nigeria (8%, 160), Poland (7%, 138), Pakistan (6%, 125), Ghana (6%, 125) and the UK (6%, 123). Timing of arrival into the UK was available for 46% (923) of the 1986 women born outside the UK, of whom 53% (489) had arrived at least 5 years prior to the pregnancy, 22% (206) 2–4 years before and 25% (228) in the 2 years before. Overall, 39% had either a basic or less than basic level of English (Table 1). Among 705 women without fluency in English and with data available on first language, this was most commonly: Chinese language (20%, 142/705), Romanian (16%, 114/705), Polish (8%, 54/705), Urdu (5%, 38/705), Albanian (5%, 36/705) and Somali (5%, 36/705). Data on family origin are shown in Table 2.

**Table 2.** Family origin (*n* = 1919)

Family origin	*n* (%)
African or African-Caribbean (excluding North Africa)	662 (34)
South Asian	255 (13)
South-east Asian	379 (20)
Other Asian family origins	37 (2)
Other non-European (including North Africa, South America, Middle East)	50 (3)
Southern and other European	479 (25)
United Kingdom	27 (1)
Northern and any other European family origin	30 (2)

Antenatal booking was at median 11.3 [IQR 9.6, 14] gestation weeks but occurred at or after 20 weeks in 10% (251/2491) of pregnancies. HBV had been diagnosed before antenatal booking in 70% (1783/2533) of pregnancies overall. Women newly diagnosed as HBsAg-positive were younger, more likely to lack basic English and more likely to have arrived in the UK within the last 2 years compared with those with a prior HBV diagnosis (Table 3). Obstetric history was associated with timing of HBV diagnosis, with 56% (467/834) of women with no previous live birth being newly diagnosed in the audit pregnancy *vs.* only 15% (253/1636) of those with at least one previous live birth (*χ*^2^ = 439.38, *P* < 0.01). Of 1439 pregnancies with approximate timing of prior HBV diagnosis, this occurred at least 5 years before the pregnancy in 45% (642).

**Table 3.** Maternal characteristics by timing of hepatitis B diagnosis

	Hepatitis B diagnosis before this pregnancy	Newly screened HBsAg-positive in this pregnancy	*p*
Median maternal age [IQR]	32 [28, 35]	29 [26, 32]	<0.01
Less than basic English	16% (243/1504)	31% (192/612)	<0.01
Arrived in the UK less than 2 years before pregnancy^a^	15% (94/624)	46% (131/284)	<0.01

Pregnancies are the denominator.

a Limited to 908 women born outside of the UK with timing of arrival and timing of hepatitis B diagnosis available.

At notification, 11.8% of pregnancies had at least one marker of higher infectivity reported, of those with data available for at least one marker from a post-conception sample (Table 4). Based on available post-conception HBV DNA data, 4.4% (57/1293) had a VL of >10^7^ IU/ml (an indication for third trimester TDF in 2013 NICE guidelines [26]) and 6.9% (89/1293) would have been eligible for treatment using the lower cut-off of >200 000 IU/ml recommended by EASL since 2017 [16]. Those with HBV DNA level of >10^7^ were younger (21% (12/57) were <25 years *vs.* 10% (230/2385) with a lower VL, *p* = 0.013 for comparison across all age groups) and more likely to be from Asia (78% (38/49) *vs.* 33% (671/2013) of the remainder, *p* < 0.01 for comparison across all regions). In the 42 pregnancies among women who were HBeAg-negative and anti-HBe-negative, maternal characteristics were similar to the remainder with respect to prior hepatitis B diagnosis, age and region of origin (data not shown); 20 of these 42 pregnancies had an HBV DNA measure available, and none of these measures were >10^7^ IU/ml.

**Table 4.** Infectivity markers reported at notification

	% (*n*) of pregnancies
HBeAg-positive	10.0% (236/2371)
HBeAg-negative and anti-HBe-negative	1.8% (42/2315)
HBV DNA ⩾1 × 10^6^ IU/ml	6.1% (79/1293)
Any one of the above	11.8% (288/2450)

Denominator for each marker is the total number of pregnancies with data on that marker from a sample taken after conception (or, for the total of 2450, for any of the three markers listed).

Overall, antiviral therapy was received in 10.8% (180/1672) of pregnancies with data available and 74% (37/50) of those with HBV DNA level of >10^7^ IU/ml (treatment data missing for seven with a high VL). Of 180 women who received an antiviral drug during pregnancy, data on the drug received were available for 171; 89% (152) received TDF and the remainder received another drug with or without TDF.

Late initiation of ANC at ⩾20 weeks gestation was more common in pregnancies among women who had less than basic English, and had arrived in the UK within the previous 2 years or had no previous HBV diagnosis. ANC was less commonly initiated late for pregnancies among women with one previous live birth than those among women with no or ⩾2 previous live births. Women from Asia and the UK were less likely to initiate ANC late than those from Africa or Europe. There was no difference in proportion booking late by infectivity markers (Table 5).

**Table 5.** Factors associated with late booking and with missing at least one specialist appointment in pregnancy

	% (*n*) booking at ⩾20 gestation weeks for antenatal care *N* = 2491 pregnancies with timing of booking available	*p* value	% (*n*) missing ⩾1 specialist appointment in pregnancy *N* = 2337 pregnancies with specialist appointment scheduled after estimated date of conception	*p* value
Maternal age (years)				
<25	13.1% (32/244)	*p* = 0.275^a^	23.6% (52/220)	*p* = 0.034^a^
25–29	10.1% (81/800)		18.9% (141/746)	
30–34	9.4% (74/784)		16.9% (123/727)	
35–39	9.4% (47/499)		17.6% (80/455)	
⩾40	10.4% (17/164)		14.5% (21/145)	
Previous live births				
None	11.9% (98/822)	*p* = 0.009	16.3% (125/767)	*p* = 0.010
1	7.5% (62/832)		17.2% (133/775)	
⩾2	10.3% (80/776)		21.9% (162/739)	
Hepatitis B diagnosis before audit pregnancy				
No	13.6% (100/733)	*p* < 0.001	21.0% (143/681)	*p* = 0.027
Yes	8.5% (149/1752)		17.1% (282/1649)	
English language				
Fluent	8.0% (102/1282)	*p* < 0.001	16.7% (198/1185)	*p* = 0.017
Basic	8.1% (31/382)		13.9% (51/366)	
Less than basic	18.3% (77/421)		21.5% (86/400)	
Region of origin^b^				
Africa	10.3% (66/643)	*p* < 0.001	18.8% (111/591)	*p* = 0.078
Asia	8.1% (58/718)		14.8% (102/691)	
Europe (excluding UK)	14.8% (84/568)		20.2% (108/536)	
UK	4.9% (6/122)		19.0% (22/116)	
Timing of arrival into UK^c^				
>10 years ago	11.1% (20/180)	*p* < 0.001	16.8% (29/173)	*p* = 0.147
5–10 years ago	3.6% (11/302)		12.2% (36/296)	
2–4 years ago	7.4% (15/203)		18.4% (36/196)	
<2 years ago	25.8% (57/221)		18.8% (39/207)	
Markers of higher infectivity for HBV				
No	10.2% (226/2207)	*p* = 0.449	18.5% (383/2075)	*p* = 0.419
Yes	8.8% (25/284)		16.4% (43/262)	

a Non-parametric test of trend.

b Americas and Oceania excluded from table and comparison due to small numbers.

c Denominator is pregnancies to women born outside of the UK only.

Missed specialist appointments were reported by maternity services in 18% (426) of 2337 pregnancies with an appointment scheduled and information available. Missed appointments occurred in 17.8% (370/2082) of pregnancies booked at <20 weeks gestation and 22.7% (47/207) of those booked late (*p* = 0.079). They were more common in pregnancies among younger women, those with two or more previous live births, where HBV had been newly diagnosed in the audit pregnancy, and where the woman lacked basic English. A similar proportion of pregnancies with and without markers of higher infectivity had a missed specialist appointment (Table 5).

Of 2542 pregnancies reported to the audit in total, 2174 ended in a live birth (2144 singleton pregnancies and 30 pairs of twins) and 18 in stillbirth (giving a stillbirth rate of 8.2 per 1000 births) – in addition, 95 miscarriages were reported and 12 terminations. The overall rate of preterm delivery <37 weeks gestation was 7.7% (164/2127) among livebirths with data available. For 243 pregnancies, the outcome of the pregnancy was not reported (98) or unknown due to the woman having moved away to an unknown location or outside England before delivery (145); for 49 pregnancies the outcome data were reported from a different Trust than the initial notification data.

## Discussion

This national audit highlights the diverse sociodemographic characteristics of pregnant women living with CHB in England, and provides some insights into the characteristics of subgroups who may be vulnerable to delayed or incomplete antenatal or HBV specialist care.

Overall, 94% of the audit population were born outside of the UK (*vs.* 27% delivering a live birth overall in England and Wales in 2014 [27]), reflecting high HBsAg prevalence in common non-UK maternal countries of birth and underscoring the importance of accessible care for migrants for the prevention of HBV VT. Within the audit population, the most common maternal countries of birth were China (14%), Romania (10%) and Nigeria (8%), countries with an estimated HBsAg prevalence in the general population of 6.1%, 3.4% and 11.2% respectively in 2016 [6] and accounting for 0.6%, 0.9% and 1.0% of all live births in England and Wales at the time of the audit in 2014 [27]. Antenatal HBsAg prevalence varies in England from nearly 0.7% in London to 0.35% in the Midlands and East, 0.28% in the North and 0.23% in the South [12], reflecting differing proportions of women from HBV-endemic countries; in this audit, 45% of HBV-exposed pregnancies were in London.

Overall, the audit population were older and more likely to be multiparous than the general antenatal population: only 10% were aged <25 years *vs.* 20% among all maternities in England and Wales in 2014 [27], and 32% had had two or more previous live births *vs.* 25% of women overall delivering a live birth [27]. This in part reflects recent arrival in the UK of women included in the audit: a quarter of migrants with data available had arrived within the last 2 years, a proportion that may be underestimated if recent migrants with insecure immigration status were over-represented in the 54% with missing data.

Older and parous women were more likely to have been diagnosed with HBV before their audit pregnancy, probably due in part to antenatal screening in previous pregnancies, or contact tracing or testing in other settings which in England includes GP services and genitourinary medicine clinics [28]. Globally, only 10% of people living with CHB are estimated to be diagnosed, varying from 2% in African to 16% in European and 19% in Western Pacific WHO regions, and from 6% in low income to 45% in high income countries [6]. In this audit, 30% of women were newly diagnosed with HBV of whom half had recently arrived in the UK; the proportion of new diagnoses among pregnant women with HBV is now lower and in 2019–20 was 22.8% [12]. Of note, a third of newly diagnosed women had a previous live birth; given that these women would mostly have acquired HBV vertically or in early childhood rather than in recent adulthood, this suggests that they had an older child with HBV exposure that had not previously been identified. These older children had probably been born outside of the UK, in countries without high coverage of HBV screening in pregnancy.

Timely and consistent ANC depends on accessibility of services alongside other dimensions such as service acceptability from social and cultural perspectives and financial, legal and institutional barriers, all of which may be different or specific for migrant women [29, 30]. NICE guidelines recommend initiation of ANC by 10 weeks gestation, with a minimum of 10 ANC contacts for nulliparous women (seven for parous women) [31], which excludes additional appointments that may be needed by women living with CHB. The audit population began ANC at median 11.2 weeks gestation, slightly later than the 10 weeks reported in the antenatal population overall in 2014–15 [32]. A tenth of women in the audit began ANC late at ⩾20 weeks (the same proportion as seen for the general antenatal population that year [32]), rising to almost one-fifth among those with less than basic English, and 26% among those arriving in the UK in the last 2 years *vs.* only 5% among those UK-born. Importantly, this could compromise receipt of antiviral treatment for women with high VLs to reduce VT risk, as well as other aspects of ANC. Our finding that only 74% of eligible women received antiviral treatment at the time of this audit in 2014–2015 needs further investigation. As HBV DNA level is the single most important factor for transmission, factors relating to lack of adherence to national/international recommendations need to be explored further.

Lack of language support has previously been associated with inequalities in access to maternity care among immigrants in the UK, alongside other factors [29]; in the audit, women with less than basic English were more likely to miss their specialist appointments. NICE guidelines recognise women having recently arrived in the UK or with English language difficulties as potentially being less likely to receive full ANC [31, 33], but robust evidence on interventions to provide effective support and improve timeliness and completeness of care is lacking [29]. Our results showed some differences by maternal region of origin (with markers of poor access to/uptake of care more common among women from Africa and Europe than Asia). Parous women were more likely to miss specialist appointments and may face additional specific barriers, for example, around lack of childcare.

Current IDPS standards specify that pregnant women with a new HBV diagnosis or an HBV infection of higher infectivity should have a specialist assessment within 6 weeks of the positive result being reported to the maternity service [34], reflecting additional needs for clinical evaluation and risk stratification with respect to TDF in the third trimester and infant HBIG, as well as counselling. Missed specialist appointments were reported for almost a fifth of pregnancies, with no difference by markers of infectivity. A higher proportion of newly diagnosed women missed specialist appointments than those with an established diagnosis (21% *vs.* 17%), and the former group were also more likely to initiate ANC at ⩾20 weeks gestation, limiting time to organise counselling and follow-up. Similarly, women newly diagnosed with HIV were more likely to start ANC late than those with an established diagnosis in 2009–14 [35]. Newly diagnosed women may therefore be at risk of both late and incomplete care.

In 2014, data collected by PHE on completion of the infant vaccination schedule and infection status at 12 months was not complete or available for a representative group. Subsequently, PHE scaled-up a national HBV dried blood spot testing service with the aim of increasing testing coverage of exposed infants [36]. This, along with surveillance of pregnancies among HBsAg-positive women within the Integrated Screening Outcomes Surveillance Service (ISOSS) from 2021, will provide the means to investigate frequency and risk factors for VT of HBV, including markers of ANC engagement explored here and their correlation with completion of the infant vaccination schedule. Assessment of adverse birth outcomes in this population was outside the scope of this audit. Robust prospective studies examining whether women with CHB have elevated risk of adverse outcomes such as preterm delivery are lacking, and the limited evidence available to date is inconsistent [37–39]; the preterm delivery rate in this audit (7.7% of livebirths) was the same as the general population in 2015 [27]. Stillbirths were reported in 8.2 per 1000 births in the audit *vs.* 4.5 per 1000 live births overall in England and Wales in 2015 [27]; stillbirth rates are higher overall among babies of Asian and black ethnicities due to multiple and poorly understood causes [40], and this is an important area for future research.

This audit is the largest study to date of pregnancies among HBsAg-positive women in England, and included the large majority nationally in 2014 (2542 *vs.* 3060 pregnancies reported to the IDPS Programme, a total which double-counted women receiving ANC in more than one place). In 194 (almost 8%) pregnancies in the audit, outcome data were reported from a different Trust to the notification data or were missing because a woman had moved, providing a minimum estimate of mobility of this population during pregnancy and highlighting challenges to continuity of care. Some information was incomplete, particularly on timing of arrival in the UK, and missed specialist appointments in pregnancy may be under-reported, as maternity units may not have been aware of these in time to report to the audit (or at all). The proportion of live births to women born outside of the UK has continued to increase (29.3% in England and Wales in 2020 [27]) and barriers to care experienced by different migrant groups require ongoing evaluation, alongside the potential impact of digital technologies during the COVID-19 pandemic on delivery of care.

This large audit of pregnant women living with CHB, conducted in a country with low HBV prevalence, provides some important information to inform service delivery and potential future research in similar settings. Findings regarding markers of engagement with antenatal and specialist care suggest that young women, those with a new HBV diagnosis, those recently arrived in the UK and with less than basic English and those with several children may face specific, and potentially intersecting, barriers in accessing care. Ongoing surveillance by ISOSS will allow further investigation of the characteristics and service use of pregnant women in England who live with CHB, as well as their management and related pregnancy and infant outcomes.

## Data Availability

Requests to access antenatal screening data are considered by the Antenatal and Newborn (ANNB) Research Innovation and Development Advisory Committee (RIDAC) within NHS England (https://www.gov.uk/government/publications/annb-screening-submitting-research-proposals/annb-screening-research-advisory-committee).
